# Tumor

**Published:** 1828-12

**Authors:** 


### TUMOR.

### *Tumor over the Orbitary Plate of the Frontal Bone.*

On the 30th of March, 1824, Dr. Malden was requested to see A. B., a patient in the Worcester Infirmary, æt. forty-five, a labourer. A few hours before, he had become insensible, with paralysis of all the muscles on the right side of the body; he had stertorous breathing, with the pupil of the eye contracted to a point, the iris motionless, and the pulse slow. He came to the infirmary a month before this time, complaining of pain and inflammation in his left eye, for which he was admitted by the surgeon. Upon examination, there was found a small fistulous opening beneath the superciliary ridge of the affected eye, through which a probe could be passed to the back of the orbit. A fortnight after his admission, he was seized with shivering, nausea, quick pulse, some degree of somnolency alternating with restlessness, hot and dry surface, and flushed face. These symptoms subsided, and he continued better up to the time of the attack on the 30th of March above described. Some white curdy matter escaped from the fistulous opening above the eye. Trepanning the frontal bone over the left orbit was proposed, but it was not done. Leeches were applied to the head; blood was also drawn from the temporal artery; and the bowels were, with difficulty, opened by strong purgatives and clysters of turpentine. He died five days after the appearance of the apoplectic symptoms.

*Examination of the body*, twenty-four hours after death.—A tumor, white and cheesy, of the size of a large walnut, lay above the left orbit, about the middle of the superciliary ridge. It appeared to have been formed between the tables of the frontal bone, which it had separated from each other, producing absorption of the cancellar structure; also of the inner table, and of the orbitary plate, so that the tumor was in contact with the eyeball beneath and the dura mater behind. The dura mater was adherent to the tumor on one side, and to the pia mater on the other, and at the place of adhesion was blackened for the space of a shilling. Immediately behind the tumor was an abscess, occupying the whole anterior lobe of the left hemisphere of the brain, and containing three ounces of fetid pus. The medullary substance of the brain around the abscess was softened, and had a yellow tint. The vessels of the brain were turgid with blood, and there were two ounces of serum in the lateral ventricles.

